# Apigenin inhibits fibroblast proliferation and reduces epidural fibrosis by regulating Wnt3a/β-catenin signaling pathway

**DOI:** 10.1186/s13018-019-1305-8

**Published:** 2019-08-14

**Authors:** Rui Jiao, Hui Chen, Qi Wan, Xiaobo Zhang, Jihang Dai, Xiaolei Li, Lianqi Yan, Yu Sun

**Affiliations:** Department of Orthopedics, Clinical Medical College of Yangzhou University, Orthopaedic Institute, Northern Jiangsu People’s Hospital, Yangzhou, 225001 China

**Keywords:** Apigenin, Epidural fibrosis, Wnt3a, Fibroblast proliferation

## Abstract

**Background:**

Failed back surgery syndrome (FBSS) is a common complication after the laminectomy. Epidural fibrosis is the major cause of lower back pain and other complications. Numerous studies have shown that apigenin (API) could treat various fibrotic diseases by regulating various signaling pathways, whereas no study has discussed whether API can inhibit fibroblast proliferation and reduce epidural fibrosis after the laminectomy by regulating Wnt3a/β-catenin signaling pathway.

**Methods:**

Human fibroblasts were cultured and treated with API in different concentrations for 24 h. CCK-8 detection and EdU incorporation assay were performed to detect cell viability and cell proliferation. Western blotting analysis was applied to detect expressions of proliferative proteins, Wnt3a, and its downstream proteins. Moreover, the Wnt3a gene was overexpressed in fibroblasts to define the relationship between Wnt3a/β-catenin signaling pathway and fibroblast proliferation. Wnt3a overexpressed fibroblasts were treated with API to verify if it could reverse the effects of API treatment. Twenty-four Sprague-Dawley rats were randomly divided into four groups. Laminectomy was performed and the rats were gavaged with different doses of API or 5% sodium carboxyl methyl cellulose (CMC-Na) solution for 1 month. The abilities of API to inhibit fibroblast proliferation and to reduce epidural fibrosis were evaluated using histological and immunohistochemical analysis.

**Results:**

CCK-8 detection and EdU incorporation assay demonstrated that API could inhibit the viability and proliferation rate of fibroblasts in a concentration-dependent manner. The Western blotting analysis revealed that API could inhibit the expressions of PCNA, cyclinD1, Wnt3a, and its downstream proteins. The overexpression of Wnt3a in fibroblasts could upregulate the expressions of proliferative proteins such as PCNA and cyclinD1. The inhibitory effect of API on PCNA, Wnt3a, and its downstream proteins was partially reversed by overexpression of Wnt3a. Moreover, the results of the histological and immunohistochemical analysis revealed that API could reduce the epidural fibrosis in rats by inhibiting fibroblast proliferation in a dose-dependent manner.

**Conclusions:**

API can inhibit fibroblast proliferation and reduce epidural fibrosis by suppressing Wnt3a/β-catenin signaling pathway, which can be adopted as a new option to prevent epidural fibrosis after the laminectomy.

## Background

Failed back surgery syndrome (FBSS) is considered as a serious condition primarily caused by epidural fibrosis after laminectomy. About 8–40% of patients suffer from FBSS, and 4–9% of patients need the second surgery [1]. Because the re-exposure of the fibrotic area is difficult and dangerous, the surgery to improve the symptomatic fibrosis usually yields notoriously poor results [2]. A large number of strategies, such as implanting special materials into the areas of laminectomy defects [3] and locally applying drugs [4], have been adopted to reduce epidural fibrosis, which have achieved a certain degree of efficacy but also have many limitations.

Numerous previous studies indicate that the epidural fibrosis primarily produced by excessive fibroblast proliferation can extend into the neural canal and adhere to the dura mater, which often result in the pain of leg and lumbago [5–7]. Recently, the use of multiple agents, such as tacrolimus [8], 10-hydroxycamptothecin [4], and tamoxifen [9] has been verified to be effective to inhibit fibroblast growth and reduce epidural fibrosis. Given the safety of various drugs, those with a significant effect on preventing fibrosis and without obvious toxicity have been constantly explored for clinical application.

Apigenin (API), a type of flavonoid, can be extracted from numerous herbs and vegetables, such as camomile, onion, and parsley. As a component of botanical for centuries, API has been used to treat many diseases. Previous studies have reported that API had anti-proliferation, anti-inflammation, and anti-tumorigenic effects [10–12]. Many studies demonstrate that the use of API could treat various fibrotic diseases, such as renal, liver, and pulmonary fibrosis [13–15]. Moreover, it has been reported that API can inhibit the proliferation of various cancer cells by suppressing the Wnt/β-catenin pathway [16]. These results revealed that API may exhibit anti-proliferative property by regulating various signaling pathways, which are likely to affect the epidural fibrosis process.

Plenty of studies have showed that the Wnt/β-catenin signaling pathway can affect various cell functions, including proliferation, apoptosis, and differentiation [17]. Moreover, activation of Wnt/β-catenin pathway could promote fibroblast proliferation [18]. Under normal conditions, β-catenin is phosphorylated by the destruction complex composed of glycogen synthase kinase-3β (GSK3β) [19]. When the Wnts stimulate the signaling pathway, the amount of Serine 9 phosphorylated GSK3β (p- GSK3β) increase, and the activity of GSK3β will be inhibited. Under this condition, β-catenin will be accumulated in the cytosol and translocate into the nucleus, which subsequently activates its target gene transcription [20, 21]. As a downstream protein of Wnt3a/β-catenin, cyclinD1 is subsequently upregulated, which can significantly promote cell proliferation [22].

However, it has not been evidenced whether API can inhibit fibroblast proliferation and reduce epidural fibrosis. In the present study, whether API can inhibit fibroblast proliferation and prevent epidural fibrosis by regulating Wnt/β-catenin pathway was investigated, which may provide a new treatment option.

## Materials and methods

### Reagent

Apigenin was provided by Sigma (St. Louis, MO, USA) and the purity of apigenin is 98%.

### Cell culture and treatment

Human fibroblasts were provided by Shanghai Cell Repository of Chinese Academy of Sciences. Fibroblasts were cultured in DMEM (Gibco, Grand Island, NY) containing 15% fetal bovine serum (Gibco) at 37 °C under 5% CO_2_. API was dissolved in dimethyl sulfoxide to prepare various concentrations of API solution. After the fibroblasts were seeded into dishes until reaching the density of about 60–80%, API in different concentrations was used to treat fibroblasts for 24 h.

### Lentivirus production and infection

The Wnt3a overexpression (Wnt3a-OE) plasmid was purchased from Ruinan biotechnology center (Shanghai, China). For lentivirus production, 293T cells were plated into a 6-well plate at a confluence of 70–90%. p-Receiver-Lv185-Wnt3a, VSVG, Rre, and Rev plasmids were co-transfected into 239T cells by the Lipofectamine 3000 reagent (Invitrogen, USA) according to the manufacturer’s instructions. The supernatant containing Wnt3a-OE lentiviruses was collected and filtered at 48 h after transfection. For lentivirus infection, the lentiviral supernatant was mixed with 10 ng/ml polybrene (Genechem, China) and applied to the fibroblasts. The second round of infection was performed after the first infection for 24 h. After the second infection for 48 h, the infected fibroblasts were selected with 1 μg/ml puromycin (Sigma, USA). The efficiency of infection was evaluated by Western blotting analysis and the fibroblasts which overexpressing Wnt3a were used for subsequent experiments.

### Cell viability

The cell viability was measured by Cell Counting Kit-8 (CCK-8) (Dojindo, Tokyo, Japan) following the instructions of the manufacturer. When the density of cells was about 60–80%, API in different concentrations (0, 10, 30, 60, 90, 120, 150 μM) was added into 96-well plates and maintained for 24 h. The fibroblasts were further incubated with 0.01 ml CCK-8 reagent at 37 °C. The absorbance values were measured with a microplate reader (Tecan) at 450 nm after 2 h, and the cell survival rate was calculated.

### EdU incorporation assay

To detach the proliferation of fibroblasts, Cell-Light KFluor555 EdU kit (KeyGEN, Jiangsu, China) was used. Fibroblasts were co-cultured with API in various concentrations (0, 30, 60, 90 μM) on 6-well plates for 24 h at 37 °C. Fifty micromolars per liter of EdU operating fluid was added into each well for 2 h. These cells were then stained with DAPI for 10 min after being treated with osmotic enhancers and fixatives. The inverted fluorescence microscope was used to obtain images at × 200 magnification.

### Western blotting analysis

After the treatment with API in different concentrations, fibroblasts were collected and lysed using the RIPA buffer (Beyotime, Hangzhou, China) on ice. Ultrasonic wave was used to treat the cell lysate before collection of the supernatants. The concentration of protein in the supernatants was measured with BCA Protein Assay Kit (Thermo, USA). Equivalent protein was separated by SDS electrophoresis gel and then transferred onto polyvinylidene difluoride membranes. After being blocked in 5% Bull Serum Albumin (BSA) in TBST for 2 h, the membranes were incubated with the primary antibodies at 4 °C overnight. The membranes were then incubated with the secondary antibodies at 4 °C for 2 h after being washed 3 times in 1× TBST for 15 min.

### Animal model and application of API

This study was approved by the Animal Research Committee of Yangzhou University. Twenty-four healthy SD male rats weighted 220–250 g were randomly divided into four groups and six rats in each group. After 1% pentobarbital sodium (40 mg/kg) was intraperitoneally injected, laminectomy was performed and the L1 vertebral plate was removed according to a previous study [4]. API was dissolved in 5% sodium carboxyl methylcellulose (CMC-Na). The rats were gavaged with API in different doses (1 mg/kg, 2 mg/kg, and 4 mg/kg) or 5% CMC-Na solution (control group) once a day for 1 month. After 1 month, the rats were sacrificed by a fatal dose of pentobarbital sodium and underwent the intracardial perfusion using 4% paraformaldehyde. The entire L1 spinal specimen, including the epidural fibrotic tissue and paraspinal muscles, was collected and fixed in the buffered formalin. The spinal specimen was then embedded in paraffin after decalcification using ethylenediaminetetraacetic acid (EDTA). The paraffin-embedded tissue blocks were used for the subsequent experiments.

### Histological analysis

Transverse sections of 4 μm were prepared using paraffin blocks from each group. After the use of a series of dewaxing methods, the sections were stained with hematoxylin and eosin (H&E) and Masson’s trichrome. Six odd-numbered sections with H&E staining were used to assess epidural fibrosis and calculate fibroblast number using a light microscope at the magnification of 40 and 400, respectively. Six even-numbered sections with Masson’s trichrome staining were observed to evaluate the density of collagen fiber using a light microscope at a magnification of 400. The Image Pro Plus 6.0 image analysis software was applied to measure the value of collagen optical density.

### Immunohistochemical staining

After the process of deparaffinization and rehydration, the antigenicity of tissue sections was activated in sodium citrate solution for 15 min at 95 °C. The endogenous peroxidase activity was blocked by 3% H_2_O_2_. The sections were then incubated with diluent anti-PCNA in a wet slides box at 4 °C for 12 h, and followed by the incubation with biotinylated anti-rabbit IgG for 2 h after being washed with PBS for 3 times. The diaminobenzidine solution was used for staining and hematoxylin was applied for counterstaining these sections.

### Statistical analysis

The data were statistically analyzed using the SPSS software (version 22.0). Student’s *t* test was used for the comparison between two groups, and one-way analysis of variance was used for the comparison between multiple groups. Data are presented as mean ± standard deviation. The value of *P* < 0.05 was regarded as statistically significant in all analyses.

## Results

### API inhibits fibroblast viability and proliferation

**Fig. 1:**
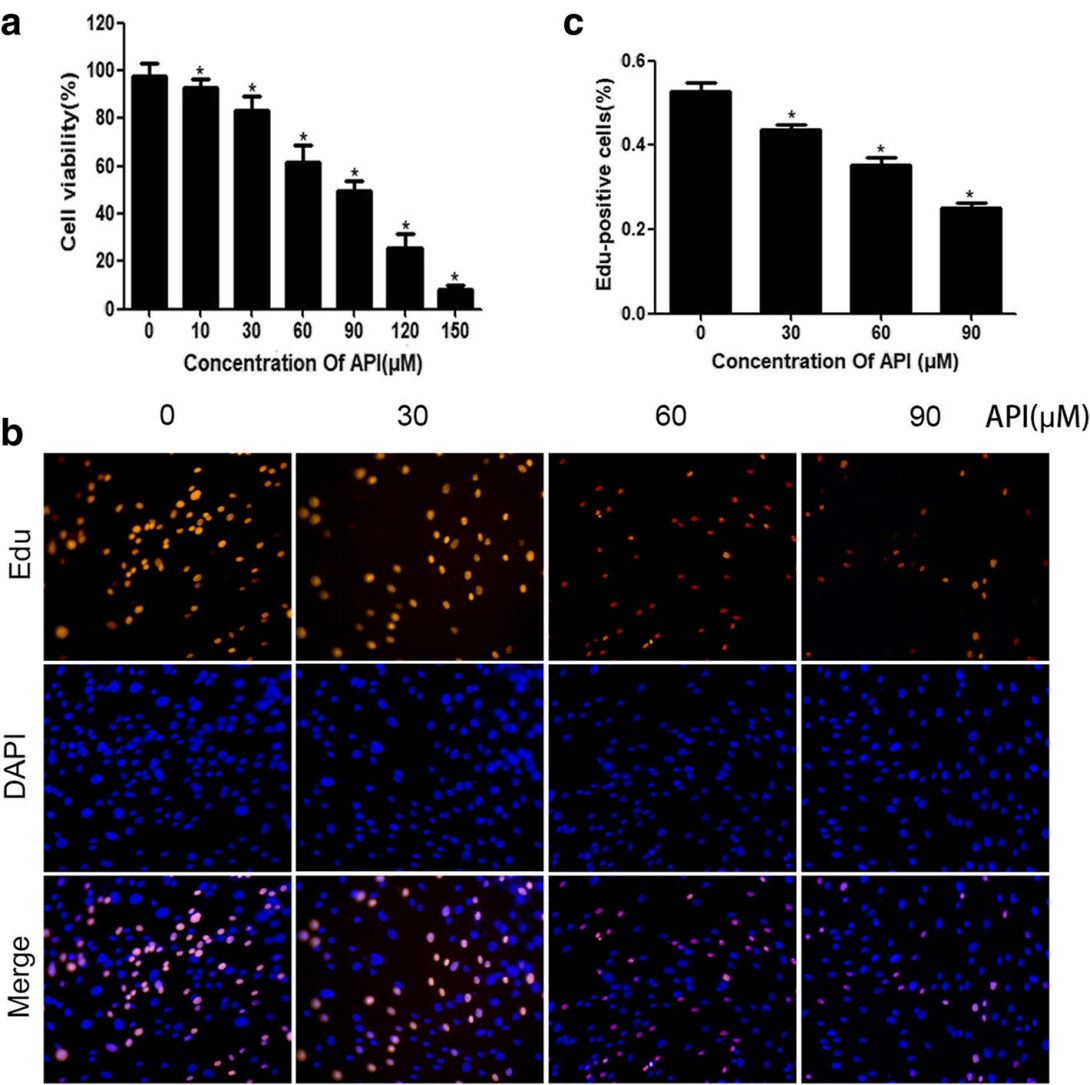
API inhibits fibroblast viability and proliferation. After treated with API in different concentrations for 24 h, the fibroblast viability was decreased in a concentration-dependent manner (**a**). EdU incorporation assay (**b**) used to detect the proliferation rate of fibroblasts demonstrated that the rate of EdU-positive cells decreased when the concentration of API increased (**c**). The columns represent the mean ± SD of three independent experiments. *P < 0.05 versus the control group

**Fig. 2:**
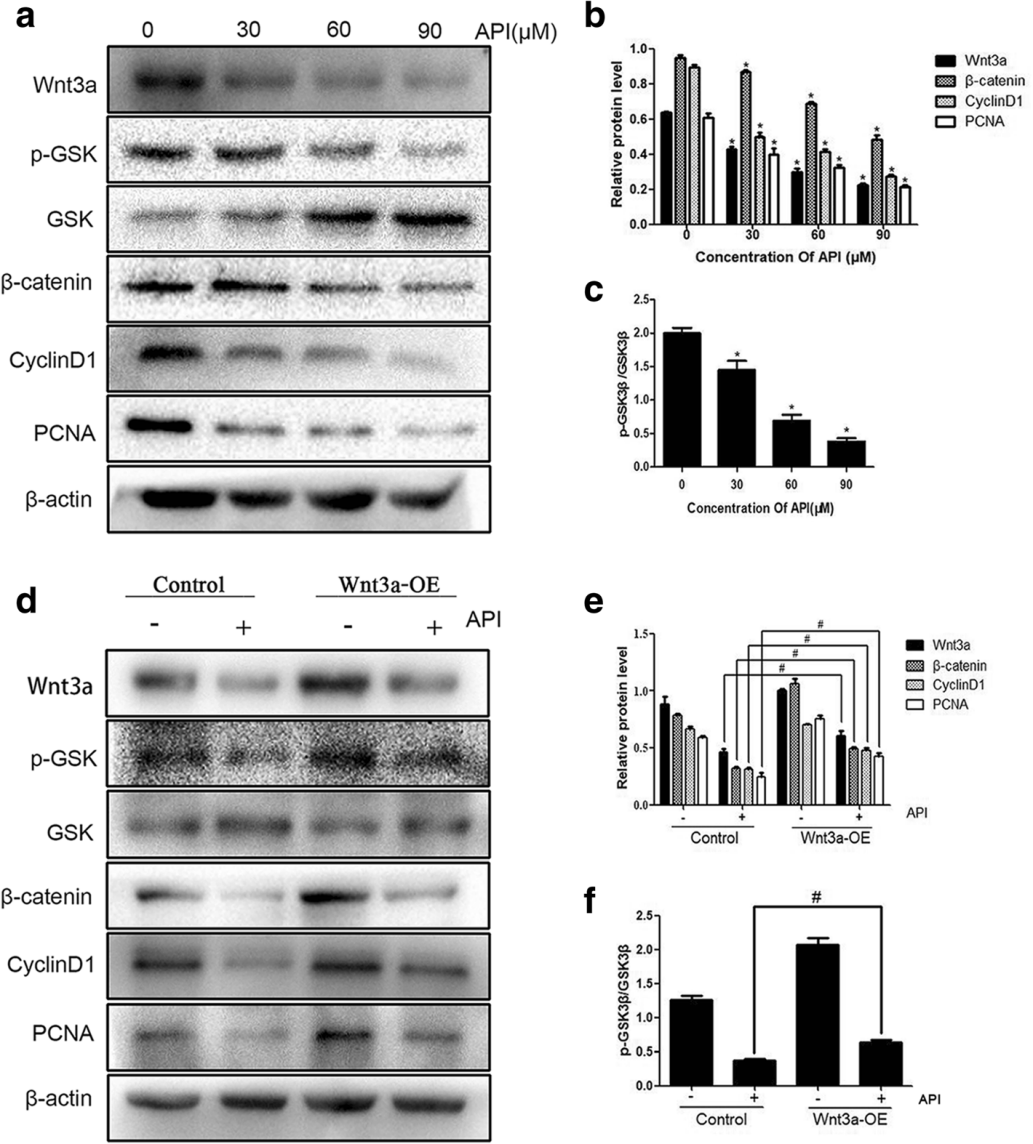
API inhibits fibroblast proliferation by suppressing Wnt3a/β-catenin signaling pathway. After the treatment with API in different concentrations, the Western blotting analysis indicated that API could inhibit the expressions of Wnt3a, β-catenin, cyclinD1, and PCNA, showing a concentration-dependent manner; the value of p-GSK3β/ GSK3β also gradually decreased (**a**–**c**). Wnt3a was overexpressed in fibroblasts and API was used to treat overexpressed fibroblasts. The Western blotting analysis showed that Wnt3a-OE promoted PCNA and the levels of Wnt3a/β-catenin related proteins expression. The inhibitory effect of API on PCNA, Wnt3a, and its downstream proteins was partially reversed by overexpression of Wnt3a (**d**–**f**). β-actin was used as a loading control. The data are mean ± SD from three independent experiments.* *P* < 0.05 versus the control group; ^#^*P* < 0.05 versus the API-treated groups

To detect the effect of API on fibroblast proliferation, API in various concentrations was used to treat the cells for 24 h. The CCK-8 detection was used to measure cell viability. As shown in Fig. 1a, API induced cell viability reduction in a concentration-dependent manner. The EdU incorporation assay was used to examine the proliferation capacity of fibroblasts. The percentage of EdU-positive cells decreased after the cells were treated with API, and the 90 μM API-treated group reached the lowest level compared with those of the other groups (Fig. 1b, c). Moreover, the Western blotting analysis indicated that API inhibited the expressions of PCNA and cyclinD1 (Fig. 2a). The results revealed that API inhibited fibroblast proliferation in a concentration-dependent manner.

### API inhibits Wnt3a/β-catenin pathway expression

To verify the effect of API on regulating Wnt3a/β-catenin pathway, Western blotting was performed to detect the expressions of related proteins after fibroblasts were treated with API in various concentrations (0 μM, 30 μM, 60 μM, and 90 μM) for 24 h (Fig. 2a). The results demonstrated that API downregulated the Wnt3a expression in a concentration-dependent manner. Moreover, API also downregulated its downstream proteins such as p-GSK3β, β-catenin, and cyclinD1 as well as the value of p-GSK3β/GSK3β, which was in accord with the reduction of cell proliferation. These results indicated that API could inhibit Wnt3a/β-catenin pathway in a concentration-dependent manner (Fig. 2b, c).

### API inhibits fibroblast proliferation by regulating Wnt3a/β-catenin signaling pathway

To further explore the underlying mechanism of how API inhibits fibroblast proliferation, Wnt3a gene was overexpressed in fibroblasts and API was then used to treat overexpressed fibroblasts. The Western blotting analysis showed that the level of Wnt3a expression was increased in Wnt3a-OE fibroblast lines, and Wnt3a-OE promoted expressions of PCNA and cyclinD1, indicating that Wnt3a could stimulate fibroblast proliferation (Fig. 2d). Upregulated expressions of p-GSK3β and β-catenin and the value of p-GSK3β/GSK3β were also demonstrated in Wnt3a-OE fibroblast lines. Moreover, the inhibitory effect of API on PCNA, Wnt3a, and its downstream proteins was partially reversed by overexpression of Wnt3a (Fig. 2e, f). Therefore, these results indicated that API inhibited fibroblast proliferation by suppressing Wnt3a/β-catenin signaling pathway.

### API reduces epidural fibrosis in rats

**Fig. 3:**
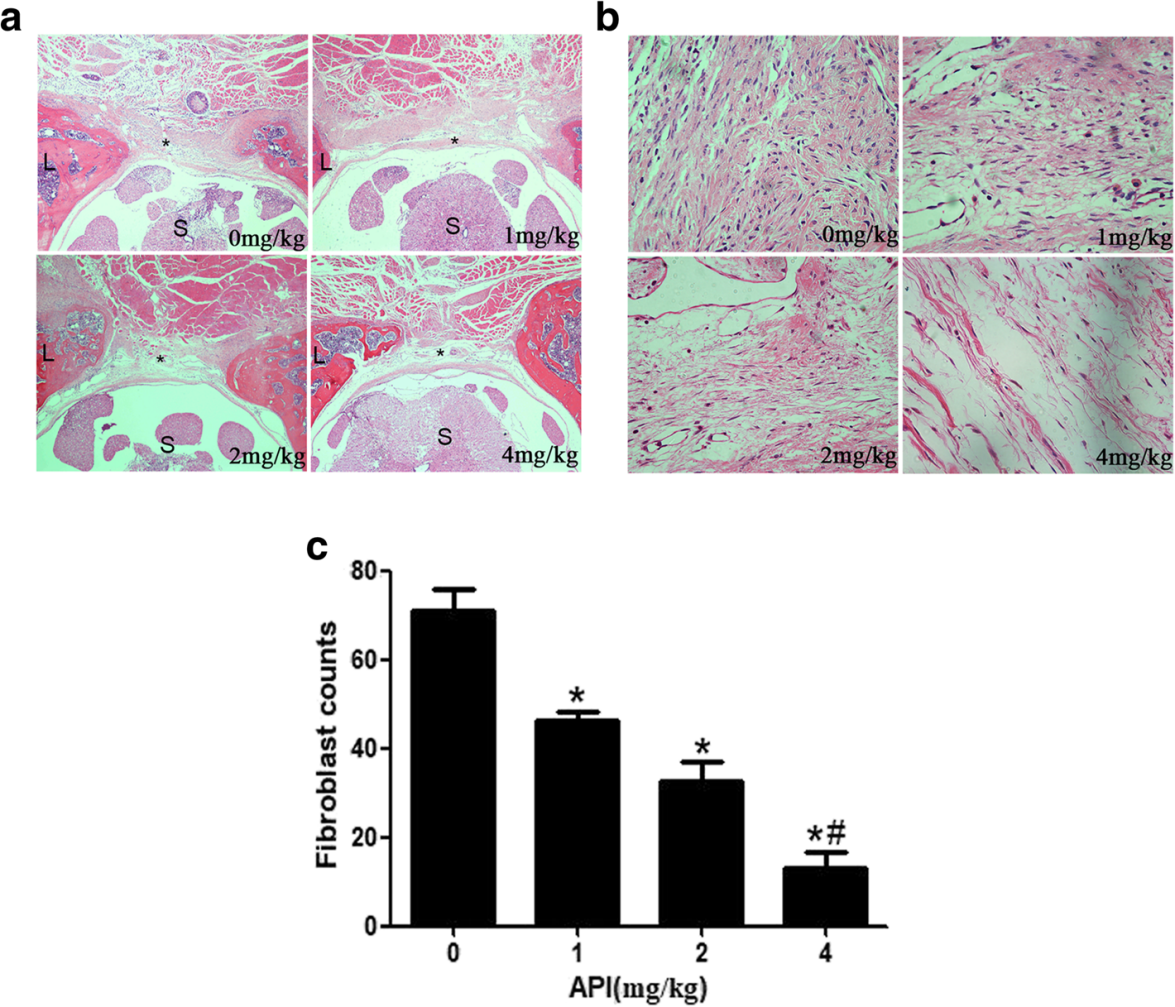
Photomicrographs of epidural fibrosis at laminectomy sites at × 40 magnification was selected from the API-treated groups and control group (**a**). Fibrotic tissue was marked as “*,” the spinal cord was marked as “S,” and laminectomy was marked as “L.” Photomicrographs of fibroblast density at × 400 magnification demonstrated a marked reduction in the API-treated groups (**b**). The number of fibroblasts is presented in the bar graph (**c**). *P < 0.05 versus the control group. ^#^*P* < 0.05 compared to the other API-treated groups

To clarify the effect of API on reducing the formation of epidural fibrosis, the laminectomy model was established and treated with API. The H&E images demonstrated that the fibrotic tissue in the surgical field was decreased after the treatment with API. Marked fibrotic tissues were observed in laminectomy sites in the control group, while loose fibrotic tissues were demonstrated in the API-treated groups (Fig. 3a). Fibroblasts were further counted from H&E images at × 400 magnification (Fig. 3b). The fibroblasts numbers in the API-treated groups were less than that in the control group, showing a dose-dependent manner. The fibroblast number in fibrotic tissue in the 4 mg/kg API group was significantly less than those in other API-treated groups and control group (*P* < 0.05) (Fig. 3c).

**Fig. 4:**
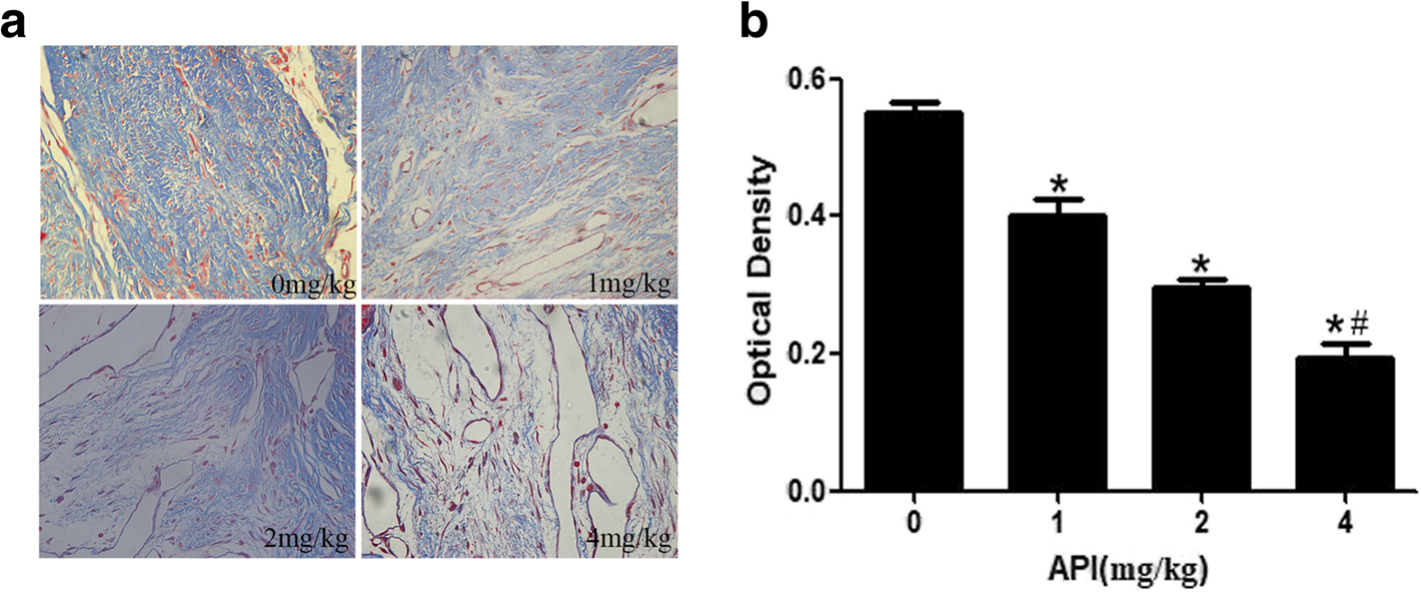
Photomicrographs of collagen density in Masson’s trichrome staining at × 400 magnification was selected from the API-treated groups and control group. As the concentration of API increased, the density of blue-stained collagen tissues decreased (**a**), and the value of collagen optical density also gradually decreased (**b**). *P < 0.05 versus the control group. ^#^*P* < 0.05 compared to the other API-treated groups

Moreover, Masson’s trichrome staining images at × 400 magnification were selected to evaluate the destiny of collagen (Fig. 4a). Dense collagen tissue was found in laminectomy sites in the control group, while weak collagen in API-treated groups. The results of collagen density in API-treated groups were coincidence with H&E staining, indicating that API decreased the destiny of collagen. The values of collagen optical density in the API-treated groups were significantly less than that in the control group, showing a dose-dependent manner. The values of collagen optical density in the 4 mg/kg API-treated group was significantly less than those of other API-treated groups and control group (*P* < 0.05) (Fig. 4b). The above results showed that API decreased fibroblast number and collagen density, which further reduced epidural fibrosis.

### API inhibits fibroblast proliferation in rats

**Fig. 5:**
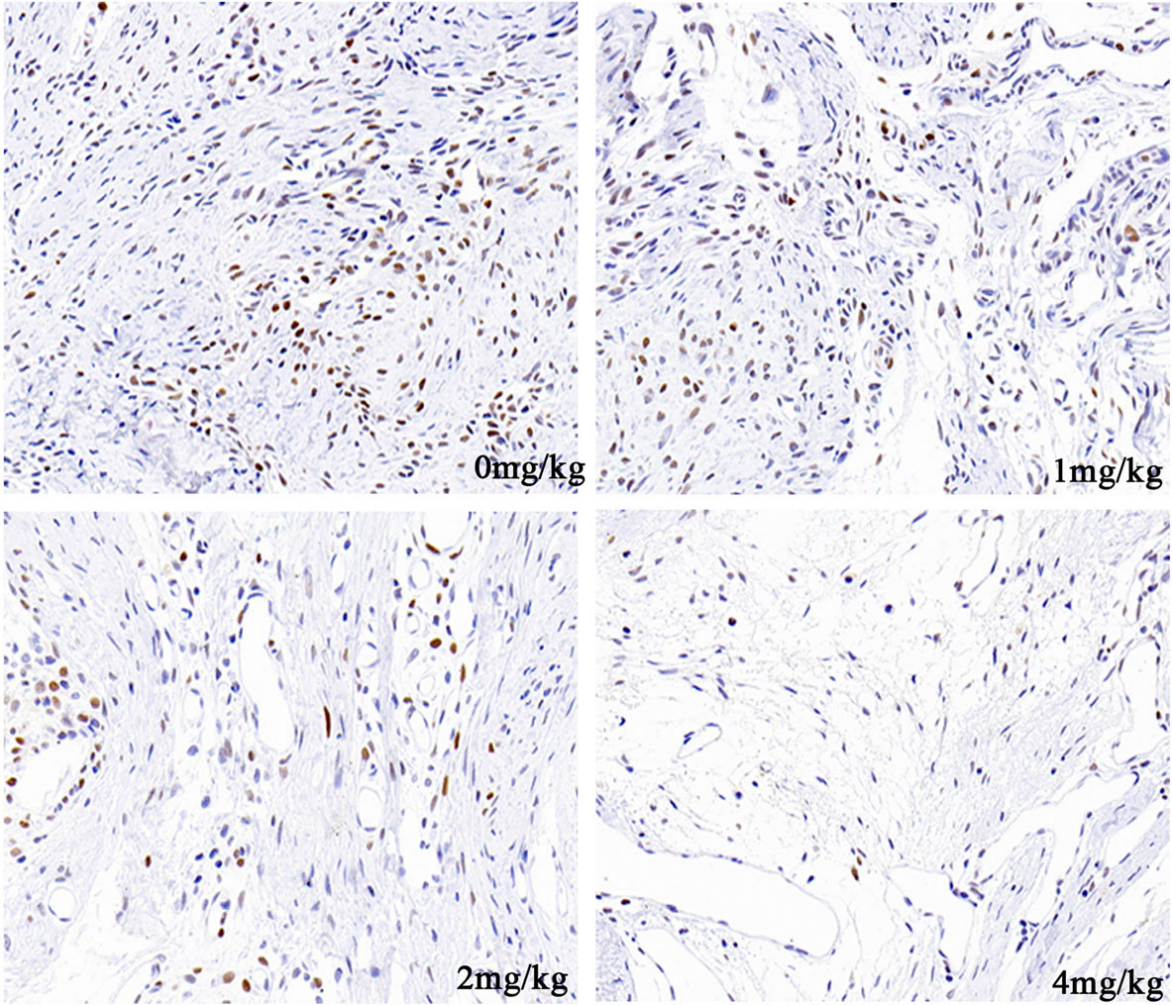
The immunohistochemical staining images of PCNA was selected from the API-treated groups and control group. Brown-stained nucleus represents PCNA-positive cells. The expression of PCNA was markedly decreased after treated with different doses of API

Proliferating cell nuclear antigen (PCNA) is a key factor in regulating cell proliferation. The immunohistochemical staining of fibrotic tissues from each treated group is shown in Fig. 5. The expressions of PCNA in the API-treated groups were lower than that in the control group. These results indicated that API suppressed fibroblast proliferation in epidural fibrotic tissue.

## Discussion

In the present study, we found that API inhibited fibroblast proliferation and reduced epidural fibrosis after the laminectomy in rats, which may be through the suppression of Wnt3a/β-catenin signaling pathway.

In in vitro study, the results of CCK-8 detection and EdU incorporation assay demonstrated that API inhibited the viability and proliferation ability of fibroblasts in a concentration-dependent manner. Moreover, the Western blotting analysis confirmed that expressions of PCNA and cyclinD1 also decreased after being treated with different concentrations of API, which was in accordance with the results of CCK-8 detection and EdU incorporation assay. These results indicated that API had an effect on inhibiting fibroblast proliferation.

In in vivo study, different doses of API were gavaged to the rats after laminectomy. Various parameters, such as histological observation, fibroblast counting, collagen density analysis, and immunohistochemical staining, were selected to assess the effect of API on reducing epidural fibrosis. Compared with the control group, looser fibrotic tissues and a lower collagen density were found in the API-treated group. Moreover, fibroblast number was decreased in the laminectomy sites after treated with API. Immunohistochemical analysis also showed that the expression of PCNA was downregulated in the API-treated groups. These results indicated that API inhibited the proliferation of fibroblasts and correspondingly reduced epidural fibrosis in rats.

API is widespread exist in fruits and vegetables that have the characters of nontoxicity and non-mutagenicity [23, 24]. Previous studies have verified that API could inhibit various cell proliferation by regulating Wnt/β-catenin pathway. For instance, API could inhibit proliferation and invasion of human osteosarcoma cells by suppressing β-catenin [25], while API reduced proliferation of human prostate cancer cells and suppressed prostate carcinogenesis in TRAMP mice by blocking β-catenin signaling [26]. However, the detailed mechanism of how API inhibits fibroblasts proliferation and reduces epidural fibrosis is unclear.

Many signaling pathways have the function that can promote cell proliferation. Previous studies have indicated that Wnt/β-catenin pathway could promote various cells proliferation in both cancer cells and non-cancer cells, such as human embryonic kidney 293 cells, human acute lymphoblastic leukemia cells, and human embryonic stem cells [27–29]. Wnt3a could downregulate the β-catenin acetylation and promoted the proliferation of human MCF7 breast cancer cells [30]. Knockdown of Wnt2 and β-catenin in human U251 glioma cells could inhibit cell proliferation and induced apoptotic cell death [31]. Moreover, it has been reported that Wnt1 overexpression promoted mouse cardiac fibroblast proliferation and enhanced expression of pro-fibrotic genes, indicating that Wnt1 enhances the pro-fibrotic function of cardiac fibroblasts and Wnt signaling pathway is closely related to organ fibrosis [32].

Previous studies have suggested that Wnt3a activated downstream signaling and stimulated fibroblast proliferation [33, 34]. In the present study, overexpression of Wnt3a in fibroblasts upregulated the expressions of proliferation-related proteins such as PCNA and cyclinD1, indicating that Wnt3a could promote fibroblast proliferation, which is in accord to previous studies. Accumulating evidence indicated that API had the ability in inhibiting cell proliferation by regulating Wnt/β-catenin signaling [35, 36]. Our study also demonstrated that API could inhibit proliferation in human fibroblasts and suppress Wnt3a/β-catenin pathway. Moreover, the inhibitory effect of API on fibroblast proliferation and Wnt3a/β-catenin pathway-related proteins was partially reversed by overexpression of Wnt3a. Therefore, the anti-fibrotic effect of API on reducing epidural fibrosis is through the suppression of Wnt3a/β-catenin signaling pathway.

During our treatment, there were no side effects such as delayed wound healing, emesis, and death, suggesting that API may be a safe drug in preventing surgery-induced epidural fibrosis after laminectomy. As a type of oral agent, API had many benefits to the gastrointestinal tract [37]. A previous study indicated that API exerted neuroprotective functions in spinal cord injury animal model [38]. However, a contrary study demonstrated that API failed to benefit the course of autoimmune encephalomyelitis and suppressed recovery from acute inflammatory damage [39]. Therefore, the safety and underlying toxicity of API on reducing epidural fibrosis should be further determined before clinical application.

## Conclusion

In conclusion, the present study demonstrated that API could reduce epidural fibrosis by inhibiting fibroblast proliferation through the suppression of Wnt3a/β-catenin signaling pathway. Thus, the use of API may be adopted as a novel treatment option to prevent epidural fibrosis after laminectomy.

## Data Availability

The datasets supporting the conclusions of this article are included within the article and its supplementary materials

## Abbreviations

API: Apigenin
CMC-Na: sodium carboxyl methyl cellulose
HE: Hematoxylin and eosin
